# Optimal timing of HIV home‐based counselling and testing rounds in Western Kenya

**DOI:** 10.1002/jia2.25142

**Published:** 2018-06-08

**Authors:** Jack J Olney, Jeffrey W Eaton, Paula Braitstein, Joseph W Hogan, Timothy B Hallett

**Affiliations:** ^1^ Centre for Health Economics & Policy Innovation Imperial College Business School London United Kingdom; ^2^ Department of Infectious Disease Epidemiology Imperial College London London United Kingdom; ^3^ Dalla Lana School of Public Health University of Toronto Toronto Canada; ^4^ Department of Medicine School of Medicine College of Health Sciences Moi University Eldoret Kenya; ^5^ Department of Biostatistics and Center for Statistical Sciences Brown University School of Public Health Providence RI USA

**Keywords:** HIV, care cascade, mathematical model, home‐based counselling & testing, treatment cascade, antiretroviral therapy, HIV testing

## Abstract

**Introduction:**

Weaknesses in care programmes providing anti‐retroviral therapy (ART) persist and are often instigated by late HIV diagnosis and poor linkage to care. We investigated the potential for a home‐based counselling and testing (HBCT) campaign to be improved through the optimal timing and enhancement of testing rounds to generate greater health outcomes at minimum cost.

**Methods:**

Using a mathematical model of HIV care calibrated to longitudinal data from The Academic Model Providing Access To Healthcare (AMPATH) in Kenya, we simulated HBCT campaigns between 2016 and 2036, assessing the impact and total cost of care for each, for a further 20 years.

**Results:**

We find that simulating five equally spaced rounds averts 1.53 million disability‐adjusted life‐years (DALYs) at a cost of $1617 million. By altering the timing of HBCT rounds, a four‐round campaign can produce greater impact for lower cost. With “front‐loaded” rounds, the cost per DALY averted is reduced by 12% as fewer rounds are required ($937 *vs*. $1060). Furthermore, improvements to HBCT coverage and linkage to care avert over two million DALYs at a cost per DALY averted of $621 (41% less than the reference scenario).

**Conclusions:**

Countries implementing HBCT can reduce costs by optimally timing rounds and generate greater health outcomes through improving linkage, coverage, and retention. Tailoring HBCT campaigns to individual settings can enhance patient outcomes for minimal cost.

## 1 Introduction

Remarkable progress has been made during the past decade towards reducing HIV‐related mortality, reducing transmission and prolonging the life‐expectancy of HIV‐positive individuals 1, 2, 3. With a 42% decline in annual AIDS deaths since the peak in 2004 and now over 19 million people on treatment across the world, the spread of AIDS has been halted and reversed 4, and the 90‐90‐90 strategy announced by UNAIDS in 2014 aims to eliminate new infections 5. However, current diagnostic programmes are not maximally effective. They often do not identify infected individuals in a timely manner, leading to the late initiation of patients onto treatment and poor patient prognoses 6, 7.

The 90‐90‐90 targets state that by 2020, 90% of all infected individuals will be diagnosed, 90% of diagnosed individuals will be on treatment and 90% of patients on treatment will be virally suppressed 5. The long‐term goal is to achieve 95% in each of these indicators by the year 2030 8. Diagnostic and treatment programmes must ensure they are able to achieve these future targets and also adapt to changing treatment guidelines. The latest WHO Guidelines on ART now suggest treatment should be given to all individuals regardless of their CD4 count 9. With an expanding inventory of interventions that variously address deficiencies in the cascade of care 10, 11, programme managers must identify the most appropriate intervention, or combination of interventions, to maximize patient outcomes cost‐effectively 12.

HIV‐testing is the first contact a patient will have with the care setting, and it is therefore crucial that care programmes maximize the effectiveness of diagnostic strategies to identify as many infected individuals as possible, because downstream losses will only diminish the number of persons actively engaged in care. Home‐based counselling and testing (HBCT) is an effective means of identifying infected individuals, as demonstrated by several successful trials in sub‐Saharan Africa 13, 14. The logistics of HBCT campaigns vary by site, but broadly, when a campaign is implemented, a trained counsellor visits houses to gather information on the occupants, provide sexual‐health information, and to those who consent, HIV‐testing and counselling 15. Crucially, as patients are visited at home, they do not have to expend any effort or money seeking diagnosis; although, following diagnosis, patients need to attend a clinic to enrol into care, and this linkage step has often presented a challenge to HBCT campaigns 16.

With HBCT programmes being implemented to various degrees in many resource‐limited settings 17, the question of how often to repeat HBCT rounds arises 18. For instance, if the entire population is covered by HBCT during a single round, then any further cases identified by a subsequent round would only be incident cases, those missed in the earlier round, or migrants; this would result in a high cost per infected patient identified, although would provide an estimate of incidence 18. The impact of transmission during acute infection on incidence is still hotly debated 19, 20. Estimates of transmission during this stage continue to vary but point to acute infection playing a disproportionate role in transmission; reinforcing the need for early diagnosis and treatment 20. Additional questions surrounding the importance of linkage to care following testing, and whether coverage or linkage should be prioritized in order to maximize patient outcomes also emerge. We utilized a previously described mathematical model of HIV care that has been calibrated to ART programme data from AMPATH in western Kenya in order to investigate these questions 12. We conducted a theoretical exploration of the determinants behind the optimal timing of HBCT rounds, before exploring how HBCT can be further enhanced to allow care systems to achieve improved patient outcomes and maximize impact for a given cost.

## 2 Methods

### 2.1 Model design

We developed an individual‐based micro‐simulation that models the entire population of Kenya, describes the HIV epidemic, and is able to make projections regarding HIV incidence and disease burden. The model simulates HIV progression and also captures the care experience of patients as they move through an ART programme following diagnosis through either voluntary counselling and testing (VCT) at a clinic, provider‐initiated counselling and testing (PICT) at a hospital, or through HBCT at home. From 2016, immediate treatment is provided to all individuals attending care 9. A detailed overview of the main characteristics, epidemiology, treatment guidelines and assumptions of the model are shown in Table 1, while further details regarding model calibration have been published previously 12.

**Table 1:** Basic model information and assumptions 12

Characteristic	Description
Population	The model simulates the entire population of Kenya from 1970 until 2056. In 1970, the age distribution of the population is matched to estimates from the United Nations (UN) 21; thereafter, annual population growth is captured through births uniformly distributed throughout the year and informed by data from the World Bank 22. Natural life expectancy is derived from mortality rates by the UN 23.
HIV epidemic	Epidemic simulated from 1970 onwards with prevalence peaking in the mid‐1990s. Prior to 2002, incidence is driven from estimates by Spectrum 24; but after 2002, the model adjusts incidence to allow ART to elicit a reduction in new infections. In addition, this allows subsequent interventions (e.g. HBCT) to indirectly reduce incidence.
Disease progression	HIV progression is described in terms of declining CD4 counts and the development of WHO stage defining conditions. This is associated with increasing hazards of HIV‐related mortality. Upon ART initiation, CD4 counts begin to reconstitute, patients recover from WHO stage conditions, and HIV‐related mortality decreases.
HIV testing and treatment	HIV testing begins in 2004 for all adults and children through voluntary counselling and testing (VCT) at a clinic, provider‐initiated counselling and testing (PICT) at a hospital, or through HBCT at home. Testing through all three modalities occurs throughout the intervention period and at the same rate for both males and females. Once diagnosed, infected individuals link to care immediately and receive a CD4 test to determine their eligibility for treatment. If found to be eligible, patients initiate life‐long ART 12.
Treatment guidelines	Beginning in 2004, ART was made available for individuals with CD4 < 200 cells/μl or WHO stage IV. In 2011 this was updated to CD4 < 350 cells/μl or WHO stage III/IV, and in 2015 to CD4 < 500 cells/μl or WHO stage III/IV 25, 26. From 2016, we assume immediate treatment will be provided to all individuals attending care, as per WHO guidelines 9.
DALY Calculations	DALYs are used to illustrate aggregate patient outcomes. Disability weights are a function of ART and current health status and were sourced from the Global Burden of Disease Study 2010 27. See Section 6 of Appendix in Olney *et al*. for further details 12.
Assumptions	Patients can be lost from all parts of pre‐ART and ART care. Declining health drives care‐seeking behaviour and leads to patients presenting to care. If symptomatic (WHO Stage III/IV), we assume patients are able to initiate treatment immediately, avoiding the need to wait for a CD4 test result. Once initiated onto treatment, we assume that 86% of patients are highly adherent, leading to immediate viral suppression 28. Finally, if lost from ART care, disease progression resumes and patients do not naturally seek and return to care 29.

### 2.2 Home‐based Counselling and Testing (HBCT)

We simulate hypothetical HBCT campaigns between 2016 and 2036, and simulate the population for a further 20 years, until 2056, to capture the accrual of health benefits resulting from the HBCT interventions. Testing through VCT and PICT continues to occur in the background while HBCT is varied. We define the reference scenario HBCT campaign as consisting of five equally spaced rounds, starting with the first in 2016, with each achieving 90% coverage of the population. We assume that 40% of diagnosed individuals are linked to care 12. Once engaged in pre‐ART care, 10% of patients are lost before receiving each CD4 test result, and a further 25% fail to return for subsequent CD4 testing 12. Upon initiating ART, 86% of patients adhere and become virally suppressed 28. 4.5% of all patients initiating ART are lost from care in the first year, and 2% per year thereafter. Once lost from ART care, patients do not naturally return 29. The reference HBCT campaign is derived from an early AMPATH HBCT implementation in Bunyala, Western Kenya, where home‐testing began in 2010 and achieved coverage of >85% of the community during its initial rounds 16, 30.

We quantify impact in terms of total disability‐adjusted life years (DALYs) averted, and cost in terms of the total cost of all care and additional ART costs relative to the cost of a scenario in the absence of HBCT (Table 1). The effectiveness of multiple rounds of HBCT is determined through the sequential impact of subsequent rounds on DALYs averted. The total cost can be broken down into individual units consisting of the cost of HIV‐testing ($10 31), pre‐ART clinic visits ($28 32), lab‐based CD4 tests ($12 32), and annual ART costs ($367 32). Most costs were based on the CHAI MATCH study 32, but also include the cost of HBCT which comprises the cost of a home‐visit ($8, based on secondary analysis of data from van Rooyen *et al*.^14^), plus the cost of a rapid HIV‐test ($10 31). All costs are in 2013 USD, and both cost and DALYs are discounted at a rate of 6% per annum.

### 2.3 Timing algorithm

To identify the “optimal” timing of HBCT rounds in the reference scenario campaign, we simulated 20 combinations of single‐round campaigns between 2016 and 2036, with an HBCT round in a different year for each. This allowed us to identify the year in which a single HBCT round yielded the greatest benefit (i.e. the round yielding the greatest increase in DALYs averted).

We then simulated 19 combinations of two‐round campaigns to identify, and implement, the second round of greatest benefit. This process was repeated until 20 rounds of HBCT were implemented in a single campaign. These results provided us with the timings of the most beneficial combinations of HBCT rounds for campaigns containing between one and twenty rounds.

We impose a limit on the number of rounds by preventing the total cost of care from exceeding that of the reference scenario campaign by more than 10%. This is because the timing algorithm increases the number of rounds in a stepwise fashion by implementing the most effective round. Therefore, rounds are not picked to fit within an upper cost limit, but the cost of a particular campaign will likely fall close to that of the reference scenario. In addition, HBCT rounds are simulated alongside existing VCT and PICT programmes, which remain unchanged.

### 2.4 HBCT permutations

We simulated eight permutations of HBCT to investigate how programmatic changes impact the timing of rounds (Table 2). We considered changes to linkage, coverage and retention. For each, it was assumed that no additional cost was associated with any changes to home‐testing. Regardless, the total cost of care in each scenario is predominantly composed of the cost of ART care 12.

**Table 2:** Details of simulated HBCT permutations

Scenario	Detail
Linkage	
Perfect linkage	100% linkage to care following testing through HBCT
Poor linkage	20% linkage to care following testing through HBCT (half of reference rate)
Coverage	
Perfect coverage	100% coverage of the population in an active year
Poor coverage	45% coverage of the population, half of the reference coverage rate
Retention	
Perfect retention	100% retention to both pre‐ART and ART care for individuals testing through HBCT
Poor retention	50% less likely to be retained in pre‐ART and ART care, relative to reference rates, following testing through HBCT
Perfect linkage & coverage	100% coverage of the population and perfect linkage to care following testing through HCBT
Perfect linkage, coverage & retention	100% coverage of the population, perfect linkage to care, and perfect retention throughout pre‐ART and ART care for individuals testing through HBCT

## 3 Results

### 3.1 Optimal timing of HBCT rounds

With five rounds of HBCT between 2016 and 2036, the reference scenario HBCT campaign averts 1.53 million DALYs at a total cost of $1617 million between 2016 and 2056. ART care represents 60% of the total cost. This results in a cost per DALY averted (ACER) of $1060 (Table S1). The five rounds are equally spaced and occur in 2016, 2020, 2024, 2028 and 2032.

**Figure 1:**
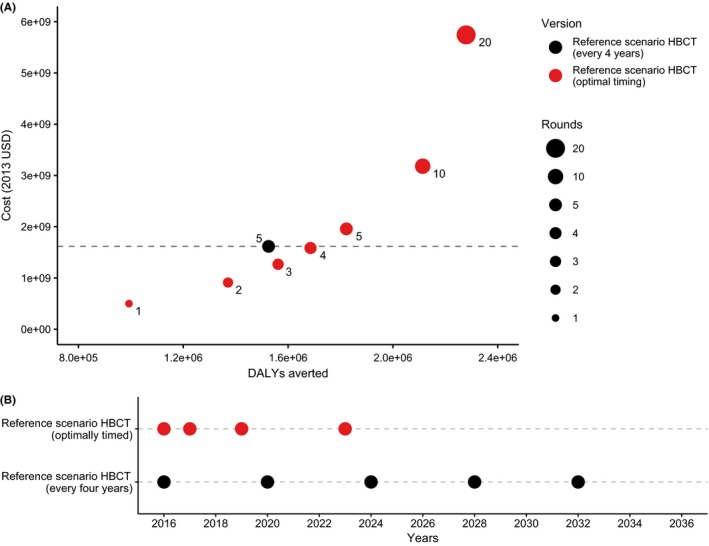
Optimal timing of reference scenario HBCT. In **(A)** four‐round campaign (red) and five‐round campaign (black) correspond to the timings displayed in **(B)**. **(A)** DALYs averted and additional cost of care for optimally timed campaigns of different sizes (red). Reference scenario campaign consisting of five equally spaced rounds (black). The number of rounds in each campaign is also shown. **(B)** Reference scenario campaign round timings. Uniformly timed rounds (black), and optimally timed rounds (red).

By simulating HBCT campaigns containing different numbers of rounds, we identified the optimal timing pattern that maximizes impact for campaigns of all sizes (Figure 1A). We find that a combination of four rounds of HBCT in 2016, 2017, 2019 and 2023 is the optimal strategy to produce maximal benefit from the reference scenario HBCT campaign (in the absence of any programmatic changes) at a cost no higher than five equally spaced rounds (Table S1). We tested for robustness by simulating minor deviations from the apparent optimal, and can confirm that our timing algorithm successfully identified the optimal position of rounds. Here, ART care again represents 60% of the total cost, indicating that four optimally timed rounds produce the same number of ART‐years as five equally spaced rounds. In addition, this optimally timed campaign averts 10% more DALYs than the reference campaign (1.69 million *vs*. 1.53 million), and the ACER is 12% less as fewer rounds are required ($937 *vs*. $1060). Whilst further increasing round size increases impact, the rate of increase per additional round decreases, as illustrated by the cost per DALY averted, which increases rapidly (Figure 1A).

By comparing the timing of four rounds of HBCT implemented between 2016 and 2036, but evaluated between 2016 and 2056, with five equally spaced rounds of HBCT, we illustrate how optimally timed rounds are “front‐loaded” to maximize the time available to accrue benefits (Figure 1B). Although, in this scenario HBCT is only able to identify and return patients lost from pre‐ART care, as we assume that patients lost from ART care never re‐engage 12, 29. Nevertheless, once all individuals are identified and linked to care by early rounds, subsequent rounds are delayed until there are sufficient numbers of incident infections to warrant HBCT again (red point in 2023, Figure 1B).

### 3.2 HBCT permutations

**Figure 2:**
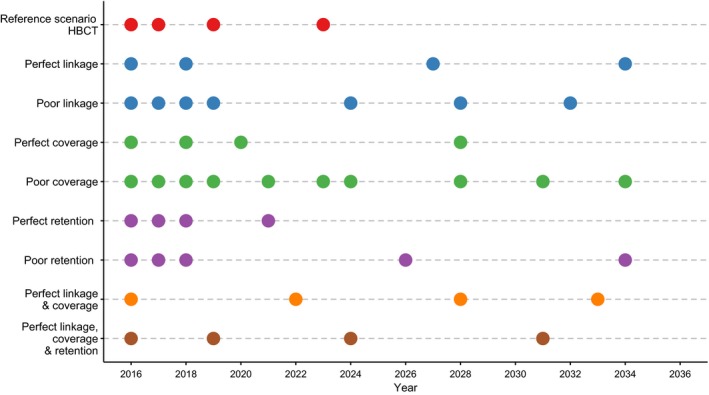
Optimal timing of HBCT rounds in various campaigns achieving the same cost produced by a uniformly timed reference scenario HBCT campaign occurring between 2016 and 2036. Poor linkage means 20% of patients link to care following testing through HBCT (half the reference linkage rate), poor coverage means 45% of the population are covered by a round of HBCT (half the reference coverage rate), and poor retention means that patients are 50% less likely to be retained in care until ART compared to the reference scenario. Full details of the scenarios described here can be found on Table 2.

Comparing the various permutations of HBCT simulated, we find that strengthening individual rounds decreases “front‐loading,” but increases the average cost per round as campaigns become more efficient at linking persons to care and hence incur greater ART costs (Figure 2). However, the total cost of all simulated campaigns does not exceed the reference scenario cost by more than 10% (Table S1). We find that in a campaign with perfect linkage, four rounds are spaced further apart than the optimally timed reference scenario campaign, but this averts 43% more DALYs (2.41 million *vs*. 1.69 million) (Table S1). In contrast, campaigns with poor linkage require more rounds to achieve the impact of the reference scenario campaign, as evidenced by the proportion of total cost represented by ART, representing a proxy for ART‐years, being similar between the two scenarios. This is because in each round, only 20% of identified patients successfully link to care following testing, whereas in the reference scenario campaign 40% link following testing. Weaker linkage in HBCT campaigns results in more pronounced front‐loading of rounds to maximize the time available to accrue benefits. In contrast, campaigns with perfect linkage exhibit rounds that are spaced further apart as each round is more efficient at getting patients to treatment (Figure 2).

During an HBCT round, the reference scenario campaign covers 90% of the population per year. Perfect coverage involves reaching the entire population in a single round of HBCT, and results in an increase in the time between successive rounds. Reducing coverage per round to 45%, substantially reduces the effectiveness of each round, and to achieve a similar impact to the five‐round reference scenario campaign without costing significantly more, HBCT with poor linkage requires 10 rounds over the 20‐year intervention period (Figure 2). Again, the proportion of total cost represented by ART care, is the same between the reference, perfect and poor coverage scenarios, indicating that for a given number of rounds, poor linkage will result in fewer individuals initiating ART in comparison to the reference scenario campaign; yet, to compensate, more rounds are required to achieve a similar number of ART‐years. Nevertheless, the major factor limiting further rounds of testing is the annual cost of ART.

If retention in pre‐ART and ART care is improved for individuals diagnosed through HBCT, we find that with perfect retention, front‐loading increases (Figure 2). This is because HBCT dramatically improves short‐ and long‐term patient outcomes, and it is therefore desirable for rounds to occur earlier to maximize the benefits of enhanced retention. With rounds in 2016, 2017, 2018 and 2021, this scenario averts 90% more DALYs than the equally spaced reference scenario campaign (2.90 million *vs*. 1.53 million), at a cost per DALY averted 43% lower ($600 *vs*. $1060) (Table S1). With poor retention in care, the first few optimally timed HBCT rounds front‐load, to bring individuals into care and maximize the time to accrue benefits, but latter rounds are pushed further apart, as retention after testing through HBCT is poorer than via VCT or PICT. As a result, five rounds are required to achieve the same ART‐time as four optimally timed reference scenario or perfect retention rounds (Table S1).

A combination of perfect coverage of the population and perfect linkage to care results in almost equidistant spacing of optimally timed rounds (Figure 2). This indicates that the timing of HBCT rounds is both a function of the efficiency of each round and time available to accrue benefit. In addition, we find that maximizing the effectiveness of each round through perfect coverage, perfect linkage and perfect retention to care, alters the timing pattern of rounds still; as the addition of perfect retention to the perfect linkage and coverage combination encourages the front‐loading of rounds due to the benefit perfect retention brings to individuals testing through HBCT (Figure 2). This perfect HBCT campaign consisting of four rounds averts 3.77 million DALYs at a cost per DALY averted of $469 (56% less than the five‐round reference scenario) (Table S1).

## 4 Discussion

This analysis illustrates that HBCT campaigns, when optimally timed and “front‐loaded,” can reduce costs without compromising health benefits for HIV‐infected individuals. In this setting, these benefits can be increased through improving linkage to care and coverage of the population, and maximized by additionally enhancing retention for patients testing through HBCT.

As care systems in resource limited settings begin implementing various forms of HBCT, it is vital to understand how this active outreach will best serve the population 14, 15, 17. We demonstrate the impact of different HBCT campaigns on patient outcomes, to understand how structural differences in campaigns alter outcomes. In populations with high rates of HIV diagnosis and low HIV incidence, HBCT campaigns may add little value unless secondary benefits, such as the return of patients who have disengaged from care, are confirmed 18. In contrast, a population with many undiagnosed individuals and steady incidence, such as populations with migrant workers, provide an HBCT campaign with more scope for impact. From a theoretical perspective, this seems logical, but in reality, it assumes knowledge of all infected individuals; something that health systems often fail to capture as they only account for individuals who have presented to care.

With over 50 HBCT programmes successfully implemented in sub‐Saharan African countries, many care systems may initiate HBCT campaigns without prior knowledge of the size of the undiagnosed population 33. Although, provided high enough coverage is achieved in the first round of HBCT, the size of the undiagnosed population can be ascertained, providing care managers with a cross‐section of care at a single point in time, and allowing subsequent rounds to be optimized based upon this care cross‐section and the effectiveness of each round.

The uniformly timed reference scenario HBCT campaign illustrates the importance of optimizing the timing of rounds to reduce costs while maintaining population health (Figure 1A). “Front‐loading” HBCT rounds is more beneficial than equally spacing them apart, as early rounds more rapidly increase ART coverage to high levels, accruing health benefits sooner than would be achieved via equally spaced HBCT rounds (Figure 1B). Furthermore, the degree of “front‐loading” increases if the time available to evaluate outcomes decreases. Nevertheless, this strategy ensures high coverage of the population and successful linkage to care, leaving future rounds to only identify incident infections. Short‐term expenditure would likely be higher in this strategy compared to the uniformly timed reference scenario, and may present challenges given the current environment of constrained resources 34. For larger impact, linkage, coverage and retention must be improved; this will likely be costly and involve active‐referral and follow‐up of patients to care 14. The cost functions in our model scale linearly with the number of patients, as described in the methods section, and therefore do not account for the additional costs associated with improving linkage, coverage and retention, aside from the increased throughput of patients. Furthermore, our results simulate HBCT campaigns over a 20‐year window (2016 to 206) and quantify impact and cost for a further 20 years (until 2056). Uncertainty in our results increases as we lengthen our time horizon, however, as the health benefits associated with of a single round of HBCT are not fully captured at the time of implementation, we feel this is an adequate compromise.

With such variability in impact across different HBCT permutations in a single setting, these results highlight the difficulties in directly comparing HBCT programmes 17. For example, 90% of diagnosed patients were linked to care during a year long pilot of an HBCT intervention in Kwazulu‐Natal, South Africa 14. This was likely due to the presence of point‐of‐care CD4 testing and follow‐up visits to ensure clinic uptake. With these additional services, the intervention was found to be highly cost‐effective 35. In contrast, an HBCT intervention with passive referral only linked 15% of newly diagnosed patients over a median of 3.4 years at an AMPATH site in western Kenya 16. The extent to which these variations in linkage are attributable to fundamental differences in HBCT structure are still not understood, but they are likely influenced by underlying differences in care‐seeking behaviour of the population as well as the differential impact of HBCT on specific risk groups.

Care‐seeking behaviour is a potential modifier of the effectiveness of HBCT campaigns, as patients must seek care following diagnosis. With evidence now indicating HBCT improves care‐seeking behaviour for common infectious disease syndromes such as acute respiratory illness 36, the impact of care‐seeking behaviour on HBCT effectiveness is still unknown and requires further research, but may in part explain the difference between these two HBCT interventions 37. In these results, the model uses individual level data from AMPATH to determine care‐seeking behaviour in a scenario where HBCT is introduced from 2010 onwards to coincide with implementation at AMPATH sites. Initial rounds at AMPATH involved only passive‐referral of patients to care, while currently implemented rounds include active referral 30. Confirmation of differences in linkage rates after active‐referral from HBCT are not yet available; however, they may be indicative of the role of care‐seeking behaviour in this setting. Further limitations of this model have been published previously 12; although it should be noted that the effectiveness of various community‐wide testing campaigns, not specifically HBCT, will depend on the characteristics explored in this paper: linkage, coverage, and retention. Therefore, while our model relies heavily on longitudinal data from AMPATH in western Kenya, our findings are likely generalizable to other community and workplace‐based, and mobile‐testing, strategies being implemented in resource limited settings.

## 5 Conclusions

The UNAIDS targets provide new goals for health systems 5. Yet, care providers must ensure their care programmes are on track to achieve these targets by their respective dates. Our results suggest HBCT campaigns, when tailored to an individual setting, optimally improve patient outcomes compared to a reference scenario campaign of evenly spaced rounds. In addition, HBCT campaign rounds can be altered to be maximally effective at a range of budget levels. Furthermore, for maximum impact, improvements to HBCT must be complemented by enhancements to other aspects of the cascade to achieve the 90‐90‐90 targets at minimum cost.

## Competing interests

JJO and JWE received grants from the Bill & Melinda Gates Foundation during the conduct of the study. TBH received grants from the Bill & Melinda Gates Foundation, World Bank, UNAIDS, Rush Foundation, Wellcome Trust, and personal fees from the Bill & Melinda Gates Foundation and WHO during the conduct of the study. JWH and PB declare no competing interests.

## Authors’ contributions

JJO, JWE and TBH developed the mathematical model, conducted all simulations, and wrote the first draft of the paper. PB and JWH oversaw the creation and analysis of the data, and provided substantial intellectual contributions on model development and interpretation of data. All authors were involved in manuscript revisions and approved the final version of the article for submission.

## Supporting information


**Table S1.** Optimal HBCT campaigns achieving maximum benefit at a cost roughly equal to that of the reference scenario HBCT campaign.
