# Supplementary material for: Optimal timing of HIV home‐based counselling and testing rounds in Western Kenya
Source: J Int AIDS Soc. 2018 Jun 8;21(6):e25142. doi: 10.1002/jia2.25142 (PMC5993164; doi:10.1002/jia2.25142)
Supplement: Supplementary file 1 — Table S1. Optimal HBCT campaigns achieving maximum benefit at a cost roughly equal to that of the reference scenario HBCT campaign. [file JIA2-21-e25142-s001.docx]

**Additional files**

**Table S1:** Optimal HBCT campaigns achieving maximum benefit at a cost roughly equal to that of the reference scenario HBCT campaign.

| HBCT Details | | Timing | DALYs averted (million) | Cost (million) | ACER |
| --- | --- | --- | --- | --- | --- |
| Reference scenario HBCT | | Five rounds, one every four years | 1.53 | $1,617 | $1,060 |
|  |  | Four optimally timed rounds | 1.69 | $1,584 | $937 |
| Linkage | Perfect Linkage | Four optimally timed rounds | 2.41 | $1,473 | $611 |
|  | Poor Linkage | Four optimally timed rounds | 1.27 | $1,532 | $1,206 |
| Coverage | Perfect Coverage | Four optimally timed rounds | 1.67 | $1,640 | $982 |
|  | Poor Coverage | Ten optimally timed rounds | 1.48 | $1,665 | $1,125 |
| Retention | Perfect Retention | Four optimally timed rounds | 2.90 | $1,740 | $600 |
|  | Poor Retention | Five optimally timed rounds | 0.88 | $1,678 | $1,907 |
| Perfect coverage & linkage | | Four optimally timed rounds | 2.47 | $1,538 | $621 |
| Perfect coverage, linkage & retention | | Four optimally timed rounds | 3.77 | $1,770 | $469 |
